# Towards Personalized Treatment Strategies for Esophageal Adenocarcinoma; A Review on the Molecular Characterization of Esophageal Adenocarcinoma and Current Research Efforts on Individualized Curative Treatment Regimens

**DOI:** 10.3390/cancers13194881

**Published:** 2021-09-29

**Authors:** Sanne J. M. Hoefnagel, Jurjen J. Boonstra, Marjolein J. A. M. Russchen, Kausilia K. Krishnadath

**Affiliations:** 1 Department of Gastroenterology and Hepatology, Amsterdam UMC Location AMC, 1105 AZ Amsterdam, The Netherlands; 2 Department of Gastroenterology and Hepatology, Leiden UMC, 2333 ZA Leiden, The Netherlands; J.J.Boonstra@lumc.nl; 3 Department of Head and Neck Surgery, Dutch Cancer Institute, 1066 CX Amsterdam, The Netherlands; m.russchen@nki.nl; 4 Department of Gastroenterology and Hepatology, University Hospital Antwerp, 2650 Edegem, Belgium; 5 Laboratory of Experimental Medicine and Paediatrics, University of Antwerp, 2000 Antwerpen, Belgium

**Keywords:** esophageal adenocarcinoma, subtyping, active surveillance

## Abstract

**Simple Summary:**

Esophageal adenocarcinoma (EAC) is one of the two major subtypes of esophageal cancer. In early disease stage, many EAC patients are asymptomatic. Most patients will present with late-stage disease in case of dysphagia and/or weight loss. Patients who undergo treatment with curative intent, have 5-year survival rates rarely exceeding 30%. Currently, curative treatment consists of chemo- and radiotherapy combined with surgical resection. Despite differences between tumors at the molecular level, all patients receive similar treatment, which results in heterogeneous therapeutic response. The aim of this review is to discuss the current research on molecular characteristics in EAC, which may predict tumor response. Moreover, we also discuss the rationale and research on adjusted regimens for EAC with for instance chemoradiotherapy and surveillance instead of (immediate) surgical resection. In future, these findings will lead to more personalized treatment approaches for EAC.

**Abstract:**

Esophageal cancers confer a major health challenge and are highly aggressive malignancies with poor prognosis. Esophageal adenocarcinoma (EAC) is one of the two major histopathological subtypes of esophageal cancer. Despite advances in treatment modalities, the prognosis of patients with EAC remains poor, with a 5-year survival rate that rarely exceeds 30% in patients treated with curative intent. Chemoradiotherapy followed by resection is the treatment of choice for EAC patients, which are deemed to be curable. Current patient stratification and treatments are based on outcomes from clinical trials. Unfortunately, the molecular heterogeneity of EAC which determines the chemo- and radiosensitivity of these cancers are not taken into account. A more personalized approach in the treatment of EAC could improve patient outcomes. This review aims at summarizing literature on translational and clinical research in the field of EAC which could be of importance to develop personalized approaches. As suggested by the TCGA, expression data features molecular classifications by different platforms, including miRNA, genomic mutations and reverse-phase protein arrays. Here, we summarize literature on transcriptomic, data-driven approaches to identify distinct subtypes of EAC associated with molecular features. These novel classifications may determine the responsiveness to chemo(radio)therapy and help to identify novel molecular targets within cell signaling pathways. Moreover, we discuss the current clinical research efforts on tailored treatment regimens for patients with EAC taking into account the heterogeneous response to chemoradiotherapy. We summarize the evidence regarding active surveillance instead of immediate surgical resection after application of neoadjuvant chemo(radio)therapy in EAC. We consider that in future patients with complete response to chemo(radio)therapy, predicted by (transcriptomic) biomarkers, might benefit most from this approach. Finally, challenges to overcome for current findings to be implemented in clinical practice and move the field forward are being discussed.

## 1. Introduction

Esophageal adenocarcinoma (EAC) is one of the two major types of esophageal cancer, besides esophageal squamous cell carcinoma (ESCC). Despite advances in both diagnostic and therapeutic techniques, the prognosis of patients with EAC remains very poor. The 5-year survival rarely exceeds 43% in patients treated with curative intent [1], and a median overall survival of merely 7.5 months has been reported in patients undergoing palliative treatment, including metastatic cases [2].

Most patients with esophageal cancer have advanced disease at the time of diagnosis, because symptoms (such as dysphagia) arise late in the course of the disease [3]. At the time of diagnosis, one out of three patients will have locally advanced tumors, that are considered for curative treatment in case patients are operable [4]. Over the past two decades, treatment has evolved from single (surgery alone) to multimodality therapy (surgery in combination with chemotherapy or chemoradiotherapy), and has become the standard of care for curative treatment. Which type of chemo(radio)therapy regimen is superior remains subject of large clinical trials.

Chemoradiotherapy (CRT) according to the CROSS regimen (radiation therapy in combination with carboplatin/paclitaxel) followed by surgical resection is the preferred curative treatment for esophageal cancer in the Netherlands and several other Western countries [5]. The mechanism of action of platinum-based chemotherapeutics, including carboplatin, oxaliplatin and cisplatin, is via formation of platinum-DNA adducts that inhibit DNA transcription, leading to cell death [6]. Taxanes, including paclitaxel and docetaxel, lead to cell apoptosis via interference with beta-tubulin and microtubule dynamics and inhibits cell mitosis [7]. 

Peri-operative chemotherapy according to FLOT (5-Fluorouracil, Leucovorin, Oxaliplatin and Docetaxel) is a frequently used alternative regimen [8,9]. Pyrimidine analogues such as 5-Fluorouracil interfere with DNA synthesis and mRNA translation [10] and this effect is enhanced by adding Leucovorin, which inhibits thymidylate synthase [11].

Chemotherapeutic regimens often used in the past include epirubicine and cisplatin, in combination with 5-fluorouracil [12]. Epirubicine induces cell death by inhibiting DNA synthesis and cleavage by targeting DNA topoisomerase [13].

Although EAC and ESCC have different pathogenesis, risk factors and geographic distributions, traditionally their treatment algorithms have been similar as these have been based on organ site and loco regional disease spread. Moreover, molecular heterogeneity within the group of patients with either EAC or ESCC potentially determines the chemo- and radiosensitivity of these cancers and is not taken into account. Current treatment strategies for EAC are conducted on the basis of empirical information from clinical trials. Unfortunately, heterogeneity in chemo-radiosensitivity and molecular characteristics between patients are not taken into account. A more personalized approach in the treatment of EAC could improve quality of life and patient outcomes [14]. 

To address the question if a personalized approach would be feasible, this review summarizes the literature on potential RNA-based biomarkers for neoadjuvant therapy response prediction in particular for EAC. Biomarkers predictive for therapy response are essential for upfront patient selection for therapy. Histopathological response to neoadjuvant chemoradiotherapy in esophageal cancer is highly variable, ranging from complete resolution of the disease to no response. Pathological response rates strongly correlate with patient survival [15,16]. The definition of pathological responders to CRT remains matter of debate in trial settings. In general clinical practice, a pathological complete response is characterized by a lack of vital residual tumor cells in both the resected primary tumor and resected lymph nodes. Alternatively, a cut-off of 10% or less remaining vital cells in the resection specimen after chemo(radio)therapy is frequently used to define responders in historic biomarker studies [17,18,19,20,21,22,23,24,25]. A pathological complete response is seen in only 23% of patients with EAC treated according to the CROSS regimen [5], and even more disappointing, patients with EAC treated with FLOT have a complete pathological response in 16% [9]. 

An important question is: should patients with EAC and a complete response to CRT undergo active surveillance instead of immediate surgical resection? Therefore, this review also discusses the rationale and current evidence and research efforts on active surveillance for EAC after CRT. Patients with a predicted poor response to CRT could be offered immediate surgical resection, to prevent unnecessary side-effects and progression of disease during CRT leading to unfavorable outcome. On the other hand, patients with a complete clinical response to CRT might benefit from surgical resection when needed in case of locoregional progression, with active endoscopic and radiologic surveillance after CRT, instead of immediate resection to potentially reduce overtreatment and related morbidity and mortality. 

## 2. Methods

We reviewed and summarized the available English literature on transcriptomic, data-driven RNA analyses to identify distinct subtypes of EAC associated with molecular features and potentially responsiveness to chemo(radio)therapy by using the snowball method and searching PubMed with key words esophageal adenocarcinoma, biomarker, RNA, sequencing, microarray, prognostic, predict, chemoradiotherapy, chemoradiation, radio- or chemosensitivity, response, outcome, survival, metastasis. Only studies using treatment naive pre-operative biopsies of EAC patients were included. A quality assessment of the included studies was performed. To answer the question on active surveillance instead of immediate surgical resection after neoadjuvant chemo(radio)therapy, we reviewed literature on this subject. The available literature on active surveillance instead of immediate surgical resection for EAC and which patients might benefit from this strategy will be summarized.

## 3. Results

### 3.1. Transcriptomic Analyses to Identify Distinct Subtypes of EAC

RNA and microRNA profiling of pretreatment biopsies and serum have shown potential for identification of predictive signatures associated with response to therapy [18,26,27,28,29,30]. 

Recently, The Cancer Genome Atlas Research Network (TCGA) performed a comprehensive molecular analysis of carcinomas of the esophagus based on somatic copy-number alterations, DNA methylation, mRNA and microRNA expression and reverse-phase protein array data [31] (Table 1). In a previous TCGA publication, gastric cancers could be classified into four subtypes on the basis of having (1) Epstein-Barr virus (EBV) infection, (2) microsatellite instability (MSI), (3) chromosomal instability (CIN) and (4) genomic stability (GS) [32]. In the analysis of esophageal cancer, EACs were shown to resemble gastric cancers more closely than ESCCs. Moreover, EACs and gastric CIN cancers formed a distinct subtype compared to the other gastric subtypes. Within the group of EAC, distinct subgroups have not been established by the TCGA. Sub analyses on EACs and gastric CIN cancers only, on the molecular data from the different molecular platforms and by integrative clustering on integrated data from all platforms, failed to show consistent segregation of EACs and gastric CIN cancers. Only clusters defined by methylation data were significantly different for EAC versus gastric CIN cancers; a higher proportion of EACs showed more frequent hypermethylation. Therefore, it was argued that EACs and gastric CIN cancers should be considered to be a single disease entity. 

Therefore, it is recommended that EACs and ESCCs should not be combined in trial settings despite their similar anatomic location. These cancers have significantly different molecular signatures. It would be more rational to investigate if EAC and gastric CIN cancer would benefit from the same treatment strategies. Unfortunately, clinical and treatment data, including clinical staging, from EACs in the TCGA cohort were not complete or not reported and association with molecular features was missing. Likely, clinical stages were very diverse as pathological stages for cancers analyzed in this study varied from stage IA to IV. Additionally, it can be assumed that treatment strategies were very diverse as the samples were obtained from a wide diversity of countries from different continents. 

Subgroup classifications in EAC based on transcriptomic profiling have been reported by several groups (Table 1). One study identified three classes of EAC by microarray profiling associated with cell turnover and genes upregulated under reflux conditions (CLDN18) and in response to bile exposure (MUC5AC), metabolic processes and immune-response pathways, respectively (Table 2). 

The first subclass was associated with the worst overall survival and the third subgroup with longest overall survival in the discovery dataset; however, this prognostic effect was only modest. A total of 24% of cases were treated with palliative intention, while curative treatment was applied for 76% of cases. Although the described collection of chemotherapy and radiotherapy-naïve patient material by either endoscopic biopsy sampling for some cases or surgical resection for others, suggests that at least part of the patients treated with curative intent did not receive neoadjuvant therapy, exact therapy regimens were not specified. One other caveat is that in this analysis only intestinal type of EAC was included [33]. 

Moreover, only adenocarcinomas at the GEJ as defined by Siewert were included. Surprisingly and in line with the reported upregulation of genes related to reflux and bile exposure, the subgroups were strongly associated with presence or absence of Barrett’s (Group 1: 93.3%, Group 2: 60.7%, Group 3: 40.9%). It should be stated though that data regarding prevalence of Barrett’s was incomplete. 

These subgroups and associated transcriptomic regulation were successfully validated in four independent datasets, including one dataset profiled by RNA sequencing. However, similar association between subgroups and overall survival was only validated in two out of four datasets.

In another cohort of 64 unique EAC patients undergoing chemoradiation (exact regimens unspecified) followed by surgical resection (most commonly performed procedure was Ivor-Lewis esophago-gastrectomy) as primary treatment, three subgroups were identified based on microarray profiling, of which the smallest subgroup was associated with poor recurrence-free and overall survival and over-representation of genes implied in metastasis, proliferation and downstream signaling of NF- kB [34] (Table 2). Validation of these subgroups in an independent cohort was not performed. However, 10 genes enriched in the poor-survival subgroup were selected as representative prognostic markers and tested for their ability to predict overall survival in an independent cohort of patients. Based on the hazard ratios from univariate Cox regression analysis on the entire cohort, they were classified as protective or risk genes. Interestingly, a subset of these 10 genes (protective genes *AKR1B10, SOX21* and risk genes *DKK3, SPP1*) overlapped with the differentially expressed genes as defined by a for multiple testing adjusted *p*-value < 0.05 between the Bornschein subgroups. 

Similarly to in Kim et al., *AKR1B10* and *SOX21* were enriched in the with worst overall survival associated group 1 from Bornschein et al., when compared to group 3 and compared to group 3 and 2 (in individual analyses), respectively. In contrast to Kim et al., in Bornschein et al. *DKK3* and *SPP1* were enriched not in poor survival associated group 1 but in group 3, with longest overall survival and enrichment for immune-response pathways. 

These genes have been investigated previously by other groups. AKR1B10 was identified as a direct target of tumor suppressor gene *p53* and overexpression has been associated with increased *p53*-induced apoptosis and inhibition of tumor proliferation in colorectal cancer [35], *SOX21* is involved in Wnt/β-catenin signaling [36]. DKK3 may function as a tumor suppressor gene [37], and SPP1 functions as a cytokine resulting in increased interferon-gamma and interleukin-12 expression, and has been associated with enhanced tumor cell invasion and dissemination in EAC [38,39].

Two genes, SPARC and SPP1, both overexpressed in patients belonging to the subgroup with poor survival in the discovery cohort, were also associated with poor overall survival in the validation cohort. SPARC is involved in extracellular matrix synthesis and changes to cell shape, which can promote tumor cell invasion [39]. SPP1 is associated with cell-matrix interaction, cell adherence and invasion [39]. Potentially, the subset of patients with upregulation of these genes and poorer survival when treated with neoadjuvant treatment followed by surgery would benefit from other treatment strategies such as immediate surgery or other therapy regimens such as immunotherapy. Unfortunately, specific genes that would be able to identify patients with very good survival after neoadjuvant treatment followed by surgery, that would potentially benefit from a surgery-when-needed strategy were not shortlisted nor validated in this study. Whether SPARC and SPP1 are epithelial or mesenchymal markers is also to be determined. Unlike the TCGA and Bornschein et al., it was not clear if samples were subjected to pathological review to assess tumor purity, or macro- or microdissection to maintain high tumor cellularity thresholds. 

Overall, the quality from the TCGA, Bornschein et al. and Kim et al. were rated +, +/− and − (Table 3). All studies were retrospective in design and were performed on a sufficient number of cases. Quality assessment of RNA was well described in all three studies. The threshold of tumor percentage for the biopsies used for analyses were described for the TCGA and Bornschein et al.; however not for Kim et al. Independent cohort validation was only performed in Bornschein et al., and only for a subselection of genes in Kim et al. 

### 3.2. Active Surveillance Instead of Immediate Surgical Resection

Several studies have demonstrated that a subset of EAC patients may have complete response to CRT. These patients have no residual tumor in their resection specimens. This notion has raised the question if organ sparing therapies and the rationale and evidence for personalizing treatment strategies, specifically in patients with a complete response to CRT, needs to be addressed. 

In EAC patients with complete response, definitive chemoradiotherapy (dCRT) might be an alternative, comparable to nCRT followed by surgery. In locally advanced ESCC, two RCTs showed no difference in survival between patients treated with dCRT versus nCRT followed by surgery [40,41]. However, for EAC, prospective trials comparing dCRT versus nCRT and surgery are lacking. There is data from observational, retrospective studies, but biases that accompany these types of studies should be taken into account. For example, active surveillance instead of immediate surgical resection is generally applied in patients unfit to undergo surgery or because of personal preferences. 

In one study, elderly patients of 70 years and over, 33 with EAC and 23 with ESCC, who were treated with dCRT and declined or were unfit for surgery, had comparable survival to patients with complete clinical response undergoing nCRT and surgery [42]. A subset of patients had interval esophagectomy after dCRT. In this selected population dCRT with the option for salvage esophagectomy when locoregional disease relapses seemed a save strategy.

In another study, on a total of 61 patients with esophageal cancer treated with CRT without immediate surgical resection, and of which 40 patients were diagnosed with EAC, the estimated 5-year overall survival was 58.1% [43]. No separate sub analyses for EAC and ESCC were performed.

This is non-inferior to patients undergoing nCRT and surgery included in the CROSS trial, for which an overall survival of 47% at 5 years was reported [44]. All of these 61 patients had a complete clinical response after CRT, defined by no cancer in endoscopic biopsies and only physiologic uptake on PETCT. Thirty-three patients developed disease relapse and 20 out of those had distant metastases. Of note, 10 patients with EAC and 3 patients with ESCC developed loco-regional recurrence, of which 12 patients underwent delayed esophagectomy. As all these patients had R0 resections, so wait-and-see policies did not negatively impact these delayed esophagectomies. 

The same group reported comparable relapse-free survival and overall survival, when comparing 36 patients with esophageal cancer (30 with EAC) treated with CRT only leading to a complete response, versus a propensity-based matched cohort of 36 patients with esophageal cancer (30 with EAC) treated with nCRT followed by immediate surgical resection [45]. Again, the patients that were initially treated with CRT only, underwent delayed esophagectomy in case of loco-regional recurrence (*n* = 11). 

Delayed esophagectomy is only possible in patients with merely locoregional recurrence, in contrast to patients with distant metastases as first presentation of disease recurrence. After nCRT and surgery, locoregional recurrence without distant metastases was seen only in 3.3–5% of patients after surveillance with diagnostics on indication only for patients from the CROSS trials in Oppedijk et al. and surveillance every 3 months for 1 year, every 6 months for 2 additional years and yearly for at least 5 years with CT or PETCT every visit and endoscopic evaluation every 6 months in the first 18 months and thereafter yearly for patients with EAC in Sudo et al. [46,47]. For dCRT, two studies on mixed EAC and ESCC reported locoregional disease without distant metastases as the primary relapse in as many as 34% and 55% of all patients with recurrent disease after surveillance with imaging or endoscopic examination on indication only [48,49]. For EAC only, a rather low percentage of 31% of cases with disease recurrence has been reported to have only locoregional disease, despite routine endoscopy with biopsying and imaging by CT or PETCT [50]. According to these data, at least 31% of EAC patients treated with dCRT followed by a wait and see strategy can potentially be offered salvage surgery in case of disease relapse. This percentage could potentially be higher by improving surveillance strategies. Of those patients with distant metastases after dCRT in mixed EAC/ESCC cohorts, 35% and 49% also had locoregional disease at presentation with relapse [48,49]. In a cohort of EAC patients, 34% was reported to have both locoregional disease and distant metastases when presented with distant metastases [50].

Recently, postponed surgical resection was investigated by the SANO-study group in patients with EAC and ESCC with good clinical response as defined by intensified surveillance at 4–6 weeks and at 11–13 weeks after completion of nCRT according to the CROSS regimen [51]. This surveillance regimen makes use of several endoscopic and radiologic tools currently available, and seems adequate in detecting patients with disease relapse early on. Clinical Response Evaluation I (CRE-I) at 4–6 weeks included upper endoscopy with bite-on-bite biopsies and endoscopic ultrasonography (EUS) to measure maximum tumor depth. The second regimen applied at 11–13 weeks after nCRT was referred to as Clinical Response Evaluation II (CRE-II), included EUS, bite-on-bite biopsies and FNA of suspicious lymph nodes, and was shown to be adequate for detection of locoregional residual disease. PETCT surveillance during CRE-II adequately detected interval metastases. 

Currently, two major trials are being conducted to investigate the safety of delayed esophagectomy in patients with EAC and complete clinical response to CRT (Table 4). 

Firstly, the SANO (Surgery As Needed for Oesophageal cancer) trial (Netherlands Trial Register NTR6803), a non-inferiority phase III stepped-wedge cluster randomized controlled trial by the SANO-study group, compares overall survival rates in 224 patients with EAC and ESCC and clinically complete response to nCRT according to the CROSS regimen at 4–6 weeks, defined by bite-on-bite biopsies, and at 10–14 weeks, as defined by the CRE-II regimen [52]. Patients will undergo either active surveillance like the CRE-II regimen at 3–12 months intervals up to 60 months after neoadjuvant CRT, with surgery when needed, or neoadjuvant CRT and standard esophagectomy [53]. This study is ongoing, and first results are expected soon. Secondly, the Esostrate trial investigates survival in 300 patients with EAC and ESCC and complete clinical response to standard of care first-line CRT regimens that will undergo surveillance and salvage surgery versus standard surgical resection [54]. Final results are expected in March 2023. 

**Table 4:** Summary of study design from prospective studies discussed in Section 3.2.

Trial	Population	Intervention	Comparison	Outcome
SANO trial [52]	224 patients with EAC and ESCC and complete clinical response to nCRT	nCRT according to CROSS and active surveillance (CRE regimen ^) and surgery when needed	nCRT according to CROSS and standard esophagectomy	* Overall survival * Clinically complete response at 4–6 weeks after CROSS (bite-on-bite biopsies) * Clinically complete response at 10–14 weeks after CROSS (CRE-II regimen ^)
Esostrate trial [54]	300 patients with EAC and ESCC and complete clinical response to standard of care first-line CRT regimens	Standard of care first-line CRT regimens (not specified) and surveillance and salvage surgery	Standard of care first-line CRT regimens and standard surgical resection	* Survival

^ CRE regimen: endoscopy with bite-on-bite biopsies and EUS with FNA of suspicious lymph nodes, PETCT at 10–14 weeks (CRE-II) and 16 (CRE-6) and 30 months (CRE-9) after nCRT. * used as bullet point

## 4. Discussion

We consider that future patients with complete response to chemo(radio)therapy, predicted by (transcriptomic) biomarkers, might highly benefit from a novel organ sparing approach. The studies reviewed above were restricted to studies on patients with EAC and studies on mixed cohorts of patients with EAC and ESCC. 

Currently, several prospective trials will probably provide us with high-quality evidence regarding safety and feasibility of implementing a more personalized approach in patients with EAC and a complete clinical response to CRT. Combining results of the SANO and Esostrate seems feasible and will increase statistical power, as they both use overall survival as the primary endpoint.

It is assumable that some of the 16–32% patients who were diagnosed with both locoregional disease and distant metastases when presented with disease relapse in observational studies on dCRT, had locoregional disease before the occurrence of distant metastases, but were too late when progression to distant metastases had already occurred because of surveillance failure [48,49,50]. Therefore, to increase the proportion of patients that might benefit from surgery after dCRT or neoadjuvant therapy followed by a surveillance regimen, careful stratification and intensified and improved surveillance of patient after dCRT is of utmost importance. A reliable regimen for detection of disease relapse after neoadjuvant CRT will be the basis for a safe surveillance and salvage surgery strategy. Whether the CRE-II regimen as proposed by the SANO-study group, including EUS, bite-on-bite biopsies, FNA of suspicious lymph nodes and PETCT, is also adequate for detection of relapse more than 14 weeks after completion of nCRT is currently being determined.

The data from the SANO and Esostrate trials are essential to improving patient outcomes in EAC, which have remained stable without major advancements since the addition of nCRT to treatment by surgical resection only. Quality of life in patients with EAC could be increased with organ-saving therapy by avoiding short- and long-term complications, including reflux-related symptoms, which frequently occur due to the changed anatomy after esophagectomy and surgical reconstruction of a gastric tube or extended gastrectomy with reconstruction with a jejunal segment according to Roux-en-Y [55]. On the other hand, frequent endoscopic surveillance and the psychological distress regarding the risk of a potential disease relapse will negatively impact quality of life in patients that will qualify for a surveillance and salvage surgery strategy.

With the identification of new molecular targets and implementation of novel therapies, the number of patients that will benefit from pharmacological and radiation therapy, with or without delayed esophagectomy, is likely to increase. Robust studies to develop tools for efficient patient stratification will be of utmost importance to personalize treatment and improve patient outcomes in the near future. 

A subset of targeting therapies with efficacy in other cancer types are currently under investigation as add-on therapies in EAC. In addition, biomarkers that can aid in the selection of patients with EAC who will potentially benefit from these targeted therapies are under investigation in hypothesis-driven research. 

Pertuzumab and Trastuzumab which are targeting the HER2/Neu receptor, could be effective in curative treatment for the 5–6% of patients with EAC that are HER2/Neu positive, for example, as defined by biomarkers detecting strong protein overexpression of HER2/Neu by IHC [56,57]. Benefits from these agents on survival in EAC by well-designed prospective RCTs is yet to be determined.

Similarly, immune checkpoint inhibitors have been shown effective in multiple cancer types by restoring T cell immune surveillance against cancer cells and are under investigation for application as add-on therapy to curative regimens in EAC [58,59]. Biomarkers detecting PD-L1 expression [60], DNA mismatch repair deficiency and high mutational burden associated with increased neo-antigen presentation to the immune system are of research interest because of their potential in patient selection for immunotherapy. However, at this point, immunotherapy is not implemented as standard of care in patients with EAC undergoing curative treatment. 

To determine upfront which patients would benefit from immediate surgery or from a wait-and-see strategy, there is a high need for biomarkers that predict response to CRT. Different platforms have been used for investigation of molecular differences between EAC. Besides RNA sequencing and other molecular platforms which showed no consistent separation of EAC and CIN gastric cancers, the TCGA group used DNA methylation profiles for cluster analyses. Four different subgroups could be identified, with association to their anatomic location. Overall, progression of DNA methylation was seen from proximal to distal CIN gastro-esophageal adenocarcinomas. This might hamper research on methylation profiles and possible association with therapy responses. CDKN2A (48%) was the most frequently inactivated tumor suppressor gene by altered methylation in EAC, but CDKN2A gene alterations at this moment are mainly investigated as a marker for progression to EAC in Barrett’s esophagus [61]. Identification of altered methylation of other tumor suppressor genes and oncogenes do show potential for response prediction to specified agents and could be of interest for future research efforts [62]. 

In this review, we specifically focused and summarized literature on predictive RNA biomarkers identified by microarrays or RNA sequencing. RNA sequencing seems to be more sensitive and accurate, and delivers a larger amount of gene expression data compared to microarrays [63]. Biomarkers can be identified with a supervised analysis, with comparison of molecular features between patients assigned to different groups based on their response to therapy. Identification of subgroups by unsupervised clustering methods, rather than predictive biomarkers identified by supervised analysis, could also aid in determining which patients would benefit from certain personalized treatment regimens.

Unfortunately, at this moment, molecular markers identified by supervised analysis or derived from unsupervised cluster analysis in EAC are lacking, and therefore the urge to identify predictive biomarkers for response to CRT according to CROSS or FLOT remains high and is an ongoing topic of research. 

Caution should be paid to results that have not been validated in independent patient cohorts, as in general, ‘omics’ analyses are highly sensitive to the quality of tissue being used and large differences may occur between batches [64]. Of importance is having high-quality, freshly preserved or frozen pre-treatment biopsies to retrieve high quality, non-degraded RNA to obtain reliable profiles. On the other hand, using FFPE material of patients for RNA profiling has major logistic advantages, including the availability of patient material from larger cohorts and easier translation of findings to the clinic. Of note, the transcriptomic analyses of esophageal cancer by the TCGA group were performed on profiles from biopsies with high tumor cell content (>60%) and low content of the stromal compartment of the tumor. This introduces an important bias, because intrinsic signals from the stromal compartment and epithelial mesenchymal crosstalk signaling, are of importance in cancer and may predict patient outcomes [65,66]. Therefore, subtyping of EAC through unsupervised analyses on transcriptomic profiles with epithelial signatures using TCGA data will be confounded by the fact that stromal signatures will be low. We state that a combination of tumor and stromal compartments should be included in RNA profiling. Epithelial and stromal RNA expression data have been used for identification of independent epithelial and stromal subtypes of cancer, both relevant for prognosis and potential application of tailored therapies in pancreatic ductal adenocarcinoma [65]. Computational and biochemical efforts, including single-cell sequencing, for deconvolution of different cells will be inevitable to increase the biological understanding of RNA signals derived from conventional bulk tissue sequencing. In the past, analyses on bulk sequencing datasets were restricted to averaged expression levels across different cell types present in the tumor. Therefore, they were subject to confounding by differences in proportions of different cell types. Currently, multiple computational techniques have become available to overcome this challenge. However, important limitations with computational deconvolution may result from certain choices made in the analytic set-up, for example not including all cell types present in the analyzed bulk tissues, and may lead to relatively high error rates. More recent analyses, by single cell sequencing, allows to identify specific tumor cell populations based on expression profiles or labeling of cell surface markers and to compare gene expression specific to certain cell types between different samples [67]. 

## 5. Conclusions

Studies using RNA expression profiles to identify subgroups of EAC with potential association with patient outcomes and presence of specific targets for therapy were evaluated. The TCGA showed that EACs and gastric CIN cancers formed a single disease entity compared to other gastric subtypes associated with respectively EBV, MSI and GS.

However, two other research groups showed the existence of three subclasses of EAC. Bornschein et al. identified three classes of intestinal type of EAC, associated with (1) increased cell turnover, presence of Barrett’s esophagus and specific gene expression in response to reflux and bile and worst overall survival, (2) metabolic processes and (3) immune-response pathways, and longest overall survival. These groups were validated in independent patient cohorts.

Kim et al. also showed three subgroups of EAC, with the smallest subgroup associated with poor survival and increased expression of genes important for metastasis, proliferation and NF- kB signaling. The other subgroups were less well described regarding pathway regulation and clinical features. In both studies, *AKR1B10* and *SOX21* were enriched in the subgroups associated with poor survival. However, heterogeneity in therapy regimens applied to included patients might hamper their potential for prediction of response to specific therapy regimens.

Besides these translational efforts on identification of subgroups, which will have to be investigated for their potential to aid in selecting patients for altered therapy strategies, clinical studies on tailored treatment regimens for patients with EAC were reviewed. The rationale for a surgery-when-needed strategy after nCRT for EAC was discussed. Evidence so far is derived from retrospective studies and therefore is of limited quality. Two major research attempts on wait-and-see strategies after nCRT in EAC, with first results expected soon, were discussed.

## Figures and Tables

**Table 1:** Summary of cohorts and methods from studies discussed in Section 3.1.

Author, Country	Patient Material, Acquisition	Tumor Type	Number Included	Number for Analysis	TNM Stages	Treatment	Definition Response	RNA Profiling	Cluster Method
TCGA, Australia, Brazil, Canada, Germany, South-Korea, Moldova, Netherlands, Poland, Russia, Ukraine United Kingdom, United States of America, Vietnam [31]	Pretreatment, fresh frozen Frozen section side for pathology control with threshold ≥60% tumor nuclei and ≤20% necrosis	Gastro-esophageal adenocarcinomas	90 ESCC 72 EAC/Esophageal GEJ 36 Indeterminate GEJ AC 63 Gastric GEJ AC 140 Fundus/Body AC 143 Antrum/Pylorus AC Not specified 13	Not specified for analysis other than gastro-esophageal adenocarcinomas included	Pathological I, II, III, IV	NA	NA	RNA Integrity ≥ 7.0 Illumina HiSeq2000 PE 75 base sequencing	Integrative clustering of platform-specific clusters SuperCluster method and Clustering of Cluster assignments based on DNA methylation, Reverse-Phase Protein Array, Somatic Copy Number Alterations, messenger RNA and micro RNA cluster results
Bornschein, United Kingdom, Germany [33]	Diagnostic endoscopy or surgical resection Chemo- and radiotherapy-naïve, snap-frozen tissue Macro- and microdissection to maintain threshold ≥70% tumor cellularity at one section by H&E and pathological review	Intestinal type EAC at GEJ (defined by Siewert classification), diffuse-type and mixed pathology excluded	84 EAC patients	61 after removing samples with enrichment of genes associated with squamous differentiation	Clinical I, II, III, IV	Curative and palliative intention, not specified	Overall survival	RNA with integrity number (RIN) > 7.0 Illumina HT12 version 4.0 beadchip kit	Mclust algorithm
Kim, United States of America [34]	Diagnostic endoscopy Untreated, fresh-frozen tissue	EAC	75 cancer samples from 64 patients	75 samples	Clinical I, II, III, IV	Chemoradiation followed by surgery as primary treatment	Recurrence free survival	RNA quality index (>7) DNA microarray technology, Illumina BeadArray Reader	Unsupervised hierarchical clustering analysis based on Pearson correlation coefficients

**Table 2:** Summary of results from studies discussed in Section 3.1.

Author	Subgroups	Association Response	Differential Expressed Genes	Pathways	External Validation
TCGA, Australia, Brazil, Canada, Germany, South-Korea, Moldova, Netherlands, Poland, Russia, Ukraine United Kingdom, United States of America, Vietnam [31]	EACs and CIN gastric cancers jointly formed a group distinct from EBV, MSI or GS tumors	NA	NA	NA	No
Bornschein, United Kingdom, Germany [33]	Three subgroups	Association with median overall survival (Group 1: 25.9 m vs. Group 2: 45.2 m vs. Group 3: 83.5 m; *p* = 0.019)	Adjusted *p*-value <= 0.05 Group 1 versus 3317 genes Group 3 versus 2243 genes Group 1 versus 2204 genes 82 candidate genes for discrimination between subtypes	Group 1 “Ribosome”, “Fatty Acid Metabolism”, “Oxidative Phosphorylation”, pathways involved in nucleic acid turnover Group 2 “Steroid Hormone Biosynthesis”, “Peroxisome”, “Primary Bile Acid Biosynthesis” and pathways involved in metabolic processes. Group 3 “Antigen Processing and Presentation”, “Chemokine Signaling Pathways”, “Natural Killer Cell-Mediated Cytotoxicity”, and pathways involved in immune-response	Subtypes confirmed successfully in all four cohorts, association with overall survival consistent in BELFAST and SINGAPORE cohorts. -OCCAMS RNASeq cohort with 154 EAC and GEJ adenocarcinomas -BELFAST Affymetrix cohort with 63 EAC -SINGAPORE Affymetrix cohort with 191 gastric cancers -Asian Cancer Research Group Affymetrix cohort with 300 gastric cancers
Kim, United States of America [34]	Three subgroups	Association with recurrence free survival (worse in cluster B)	*p* < 0.002 and 1.5-fold differences between the groups cluster A versus B 2344 genes cluster B versus C 1489 genes 452 overlapping genes of the 2344 genes and 1489 genes, from which 10 selected for validation	Putative networks listed top network associated with over-representation of NF-kB	10 genes tested with qRT-PCR on RNA from FFPE in 52 EAC *SPARC* and *SPP1* associated with poor overall survival

**Table 3:** Quality assessment of studies on transcriptomic analyses to identify distinct subtypes of EAC.

Author	Retrospective or Prospective	Number Independent EAC Cases	Quality Assessment of RNA	Tumor Percentage	Independent Cohort Validation	Overall Recommendation Level (+, +/−, −)
TCGA, Australia, Brazil, Canada, Germany, South-Korea, Moldova, Netherlands, Poland, Russia, Ukraine United Kingdom, United States of America, Vietnam [31]	Retrospective	72	RNA Integrity ≥ 7.0	≥60% tumor nuclei	No	−
Bornschein, United Kingdom, Germany [33]	Retrospective	84	RNA integrity number (RIN) > 7.0	≥70% tumor cellularity	Yes	+/−
Kim, United States of America [34]	Retrospective	64	RNA quality index (>7)	NA	Only for sub-selection of genes	−
